# Estimation of antimicrobial activities and fatty acid composition of actinobacteria isolated from water surface of underground lakes from Badzheyskaya and Okhotnichya caves in Siberia

**DOI:** 10.7717/peerj.5832

**Published:** 2018-10-25

**Authors:** Irina V. Voytsekhovskaya, Denis V. Axenov-Gribanov, Svetlana A. Murzina, Svetlana N. Pekkoeva, Eugeniy S. Protasov, Stanislav V. Gamaiunov, Maxim A. Timofeyev

**Affiliations:** 1 Irkutsk State University, Irkutsk, Russia; 2 Baikal Research Centre, Irkutsk, Russia; 3 Institute of Biology of the Karelian Research Centre of the Russian Academy of Sciences, Petrozavodsk, Karelia, Russia

**Keywords:** Actinobacteria, Caves, Natural products, Fatty acids

## Abstract

Extreme and unusual ecosystems such as isolated ancient caves are considered as potential tools for the discovery of novel natural products with biological activities. Actinobacteria that inhabit these unusual ecosystems are examined as a promising source for the development of new drugs. In this study we focused on the preliminary estimation of fatty acid composition and antibacterial properties of culturable actinobacteria isolated from water surface of underground lakes located in Badzheyskaya and Okhotnichya caves in Siberia. Here we present isolation of 17 strains of actinobacteria that belong to the *Streptomyces*, *Nocardia* and *Nocardiopsis* genera. Using assays for antibacterial and antifungal activities, we found that a number of strains belonging to the genus *Streptomyces* isolated from Badzheyskaya cave demonstrated inhibition activity against bacteria and fungi. It was shown that representatives of the genera *Nocardia* and *Nocardiopsis* isolated from Okhotnichya cave did not demonstrate any tested antibiotic properties. However, despite the lack of antimicrobial and fungicidal activity of *Nocardia* extracts, those strains are specific in terms of their fatty acid spectrum. When assessing fatty acid profile, we found that polyunsaturated fatty acids were quantitatively dominant in extracts of *Nocardia* sp. and *Streptomyces* sp. grown in different media. Saturated fatty acids were the second most abundant type in the fatty acid profile. It was due to palmitic acid. Also, a few monounsaturated fatty acids were detected. The obtained materials can become a basis for development of approaches to use bacteria isolated from caves as a biological sources of bioactive compounds to create medical and veterinary drugs.

## Introduction

The rapid rise of antibiotic resistance to current antibiotics amongst pathogenic bacteria represents a large-scale issue in the field of modern medicine and healthcare (Stanton, 2013; World Health Organization, 2015). There are great difficulties in medical treatment of hospital-acquired infections both in developed and developing countries, which leads to increase of morbidity and spread of mortality (Khan, Ahmad & Mehboob, 2015). Multiple antibiotic resistance is observed in different pathogenic microorganisms such as methicillin-resistant *Staphylococcus aureus*, *Enterococcus* spp., *Enterobacteriaceae*, *Pseudomonas aeruginosa*, *Acinetobacter* spp., etc. (Magiorakos et al., 2012). Moreover, treatments become more complicated due to low efficacy of existing antibiotics and a limited number of developments of novel chemically synthetized therapeutic agents (Grabowski & Schneider, 2007; Grabowski & Kyle, 2007).

Natural products are still referred to promising biotechnological and pharmaceutical agents for development of and potential new drugs (Newman & Cragg, 2016). It is known that actinobacterial sources are estimated as about 45% of all microbial bioactive metabolites with 7,600 of these compounds (80%) being produced by the *Streptomyces* species (Hamedi, Poorinmohammad & Wink, 2017). Hence, actinobacteria are a rich and tremendous source for screening of novel metabolites with potential pharmaceutical applications (Goodfellow & Fiedler, 2010; Bérdy, 2012). Along with synthesis of natural compounds, actinobacteria are also an important source of fatty acids. Composition of cell-wall fatty acids is used for chemotaxonomy of actinobacteria. It also plays a vital role in resistance of pathogenic actinobacteria as a protection mechanism during therapy (Hamedi, Poorinmohammad & Wink, 2017).

Even though *Streptomyces* species undergo a complex life cycle with distinctive developmental and morphological stages (Hamedi, Poorinmohammad & Wink, 2017; Bentley et al., 2002), the cell wall is like that of other Gram-positive bacteria, composed of a simple peptidoglycan mesh surrounding the cytoplasmic membrane. By contrast, cell walls of *Mycobacterium* spp., as well as species of related genera including *Corynebacterium, Gordonia, Nocardia* and *Rhodococcus*, are formed by a thick meso-diaminopimelic acid-containing peptidoglycan covalently linked to arabinogalactan, which is in turn esterified by long-chain a-alkyl, b-hydroxy fatty acids called mycolic acids (Brennan, 2003; Schaechter, 2009). Also, a number of fatty acids possess both biological and antibiotic activity, such as pteridic acids, which induce formation of adventitious roots in hypocotyl of kidney beans and are produced by strain of *S. hygroscopicus*. Another example of a fatty acid with biological activity is presented by antifungal antibiotic clethramycin, etc. (Singh, Gupta & Passari, 2018; Igarashi et al., 2003).

Besides, actinobacteria are able to produce various types of biosurfactants that have antibacterial activity (Seghal Kiran et al., 2010) and play an important role in bioremediation (Alvarez et al., 2017). However, the secondary metabolism of actinobacteria is still underexplored. By virtue of genome analysis, it was estimated that actinobacteria have a number of cryptic biosynthetic gene clusters. Thus, this phylum can produce considerably higher numbers of lucrative secondary metabolites than it was expected during traditional screenings of secondary metabolites (Bachmann, Van Lanen & Baltz, 2014; Derewacz et al., 2014a; Undabarrena et al., 2017). However, the approach of one strain-many compounds (OSMAC) is still relevant for discovery of new natural products (Rateb et al., 2011a; Hewage et al., 2014). Using different production media and conditions for cultivation of microorganisms, it is possible to activate expression of some silent biosynthetic genes (Rateb et al., 2011b).

Originally, actinobacteria were known as a group of soil microorganisms producing a number of biologically active compounds. However, biotechnological capacity of classical or terrestrial environment microorganisms is reduced or exhausted in the light of the rising problem of antibiotic resistance, descending trends of classical screening new biologically active compounds, and low level of successful clinical trials of new drugs. According to the main classical hypothesis, the problem of antibiotic resistance can be partially solved by screening of novel and unstudied sources for isolation of novel microorganisms, their metabolic pathways and use of modern approaches of molecular biology, and, as a result, new natural products with biological activity (Bérdy, 2012).

Exploration of unusual and extreme ecosystems and habitats is one of the most promising ways for screening and isolation of rare strains of actinobacteria. These studies may increase the frequency of revealing new chemical molecules with biological activity, hence, development of novel medicines (Hamedi, Mohammadipanah & Ventosa, 2013; Yuan et al., 2014; Liao et al., 2016). Extremophilic microorganisms have specific mechanisms of adaptation to extreme conditions by producing unique secondary metabolites that promote their survival (Sánchez et al., 2010).

One of representative examples of unusual and extreme ecosystems is ecosystem of ancient caves rich with microorganisms (De Lurdes & Enes Dapkevicius, 2013; Man et al., 2015; Lavoie et al., 2017). Caves are nutrient-limited ecosystems characterized by stable temperatures, relatively high humidity coupled with oligotrophic conditions, and the absence of light (Northup, Kathleen & Lavoie, 2001; Schabereiter-Gurtner et al., 2002). As it was mentioned in a number of studies, actinobacteria is one of the dominant groups of microorganisms among cave microbial communities in different underground environments (Herzog Velikonja, Tkavc & Pašić, 2014; Wu et al., 2015; Lavoie et al., 2017). However, both microbial diversity of Siberian caves and biotechnological potential of natural products produced by those bacteria are still underexplored. There are several ancient caves located in Siberia that are characterized by low temperatures, great length, and long history. Previously, we published materials describing some biotechnological properties of several actinobacteria strains isolated from moonmilk speleothem collected in Bolshaya Oreshnaya cave (Axenov-Gribanov et al., 2016). In this study, we focused on preliminary estimation of fatty acids composition and antibacterial properties of culturable actinobacteria in water surfaces of underground lakes located in Badzheyskaya and Okhotnichya caves. We hypothesized that the microbial community of Siberian caves is an underexplored source for screening of new antibiotic-producing microorganisms such as actinobacteria, and can be a promising source for development of novel natural products.

## Material and Methods

### Cave description, sampling sites, and isolation of actinobacteria

The karstic cave Badzheyskaya is located near Stepnoy Badzhey village in Krasnoyarsk region (55°14′32″N 93°46′32″E) (Dublyanskiy, 1979; Ananyeva, Ananyev & Zadisensky, 2013). Badzheyskaya cave is the largest enclosure of conglomerates in the world formed in the Quaternary period. Badzheyskaya cave shows a great number of peculiar and various passages, groths and galleries and it has an underground lake and siphons. There is an abundance of clayey substances, peddle stones, and blocks of conglomerates. The cave has sparse speleothems including a small number of moonmilk speleothem, stalactites, and stalagmites. The known dimensions of the Badzheyskaya cave is 6,000 m in length, 170 m deep and 170 m wide. The average annual temperature in the cave fluctuates from 3 to 5 °C (Khizhnyak et al., 2003).

Okhotnichya cave is a karstic cave located in the Irkutsk region (52°8′18″N; 105°27′49″E). The cave length is 5,700 m. The amplitude of the cave is 77 m (Osintsev, 2010). Okhotnichya is the third in length among the known caves in Baikal region (Bazarova et al., 2014). It was formed in the Upper Proterozoic era. The cave is described by a variety of galleries and multifarious formations, namely stalactites, stalagmites, corallite, cave pearls, red-brown clays, and also, a great number of osteological remnants. Moreover, there are three small ponds and a longstanding glacier in the cave. The average annual temperature is 1.26 °C. Also, bats were found in the cave (Klementyev, Korshunov & Osintsev, 2007). Table 1 gives a brief comparative description of the studied caves.

**Table 1:** Brief comparative description of Badzheyskaya and Okhotnichya caves.

	Badzheyskaya cave	Okhotnichya cave
Type	Karstic	
Length, m	6,000	5,700
Amplitude, m	170	77
Depth	170	77
Average temperature, °C	3–5	1.26
Period of formation	Paleozoic era (Ordovic)	Upper Proterozoic era
Year of discovery	1964	2006
Current recreational load	Low	High

For actinobacteria isolation, 10 mL of water from surface of underground lakes in Badzheyskaya and Okhotnichya caves were collected in triplicate by sterile syringes in 2014. The obtained samples were transported to the laboratory in thermostatic conditions (3–5 °C), where we added equal volume of sterile 40% glycerol to each. The obtained samples were stored at −20 °C before isolating the strains.

Actinobacteria strains were isolated by triplicate plating 100 uL of each water samples on solid nutrient media. To isolate the actinobacteria strains, we used MS mannitol soy flour agar (soy flour –20 g, D-mannitol –20 g, agar –20 g, tap water –1 L, pH 7.2) (Kieser et al., 2000) supplemented with the antibiotics cycloheximide (50 ug/mL) and phosphomycin (100 ug/mL). Aliquots of collected samples (500 uL) were preheated for 5 min at 50 °C to activate spore germination and inactivate vegetative cells of other bacteria. The plates were incubated for 30 days at 28 °C and assessed for appearance of actinobacterial colony every day. Actinobacteria-like strains were selected based on colony morphology: solid density of colonies, growth inside of the agar media and steady border of the colonies (Kieser et al., 2000). The colonies were transferred from the primary plates to the fresh MS plates. Pure cultures were obtained for all colonies identified as actinobacteria on the primary plates. Several isolated strains were deposited in the Russian Collection of Agricultural Microorganisms (RCAM), St. Petersburg, Russia (Act 471/12 of 15.12.2017).

### 16S rRNA gene sequencing and phylogenetic analysis

For isolation of total DNA, strains were grown in 10 mL of TSB medium at 28 °C for 3 days at 180 rpm. Total DNA was isolated using the salting out procedures as described in (Kieser et al., 2000). To identify the isolates, the 16S rRNA gene was amplified by PCR with the actinobacteria-specific and universal primers. Actinobacteria-specific primers were: F-Act-235(CGC GGC CTA TCA GCT TGT TG) and R-Act-878(CCG TAC TCC CCA GGC GGG G) (Stach et al., 2003). Universal eubacterial primers included: 8F (AGA GTT TGA TCC TGG CTC AG) and 1492R (TAC GGY TAC CTT GTT ACG ACT T) (Shieh, Martin & Millar, 1998). The PCR reaction was performed using the ScreenMix 5X PCR kit (Kat. PK041L, Evrogen, Russia). PCR was performed in a TGradient Thermocycler (Biometra, Göttingen, Germany) in the volume of 25 uL. The PCR parameters were as follows: initial denaturation at 95 °C for 5 min, followed by 25 cycles of 95 °C for 40 s, 49–52 °C for 25 s, and 72 °C for 110 s, and final elongation at 72 °C for 5 min.

The PCR products were purified using QIAquick Gel Extraction Kit (Qiagen, Venlo, The Netherlands) and sequenced with the use of actinobacteria-specific or universal primers. Mixture of PCR product with amplification primers were sent to the Syntol company (Moscow, Russia) to sequencing of PCR products by Sanger methods (Sanger, Nicklen & Coulson, 1977). Forward and reverse sequences were assembled with Bioedit software (version 7.2.5). The obtained sequences were deposited in the GenBank with the following numbers: MG971344–MG971360 (Table 2) and aligned with the bacterial 16S rRNA gene sequences from the EZtaxon database (Kim et al., 2012; Table S1).

**Table 2:** Actinobacteria strains isolated from water of underground lakes in Badzheyskaya and Okhotnichya caves.

Cave	Strain	Accession number of isolates in NCBI database	Close strains	Accession number of isolates in NCBI database	Identity, %	Query cover, %
Badzheyskaya	*Streptomyces sp. IB2014I88-1*	MG971353	*Streptomyces globosus strain T30*	KU324456.1	100	100
			*Streptomyces flavogriseus strain SN1301-1-14*	KT597554.1		
			*Streptomyces sp. MOLA 1600*	KM274042.1		
	*Streptomyces sp. IB2014I88-2HS*	MG971351	*Streptomyces sp. strain W30*	KY402241.1	100	100
			*Streptomyces atratus strain T11*	KU324451.1		
			*Streptomyces yanii strain BCCO 10*	KP718604.1		
	*Streptomyces sp. IB2014I88-2*	MG971352	*Streptomyces sp. S1B 16S*	KF939599.1	100	100
			*Streptomyces sp. 30G*	KF772625.1		
			*Streptomyces yanii strain HHI1*	KJ573062.1		
	*Streptomyces sp. IB2014I88-3HS*	MG971349	*Streptomyces sp. MOLA 1610*	KM274041.1	99	99
			*Streptomyces sp. JSM 147831*	KR817782.1		
			*Streptomyces cyaneofuscatus strain CB2J7*	KJ531615.1		
	*Streptomyces sp. IB2014I88-3*	MG971350	*Streptomyces sp. 25BA11Y12*	KF366674.1	99	99
			*Streptomyces atroolivaceus strain 3H1*	KF554170.1		
			*Streptomyces atroolivaceus strain Ca709*	KF317994.1		
	*Streptomyces sp. IB2014I88-4HS*	MG971348	*Streptomyces scabiei isolate ID01-16c*	DQ861638.2	95	99
			*Streptomyces scabiei strain RL-34*	NR_025865.2		
			*Streptomyces sp. strain NLSt2*	KX950889.1		
	*Streptomyces sp. IB2014I88-4*	MG971354	*Streptomyces deccanensis strain QY-3*	MG751325.1	96	100
			*Streptomyces sp. strain KL33*	MG575211.1		
			*Streptomyces neyagawaensis strain ATCC 27449*	NR_025868.2		
	*Streptomyces sp. IB2014I88-6HS*	MG971346	*Streptomyces sp. strain USC-16007*	MF773763.1	100	100
			*Streptomyces sp. strain JXJ 0170*	KY613504.1		
			*Streptomyces lunaelactis strain 12L*	MG009011.1		
	*Streptomyces sp. IB2014I88-6*	MG971347	*Streptomyces sp. strain USC-16024*	MF773780.1	97	99
			*Streptomyces sp. strain USC-16014*	MF773770.1		
			*Streptomyces nigrescens strain USC008*	KX358631.1		
	*Streptomyces sp. IB2014I88-7*	MG971345	*Streptomyces sp. ACT4(2014)*	KJ187410.1	95	98
			*Streptomyces pratensis strain EA5*	KU973961.1		
			*Streptomyces cavourensis strain xsd08096*	FJ481053.1		
	*Streptomyces sp. IB2014I88-8*	MG971344	*Streptomyces anulatus strain TCA20000*	KC462526.1	100	100
			*Streptomyces sp. QLS20*	JQ838127.1		
			*Streptomyces sp. QLS56*	JQ838100.1		
	*Nocardia sp. IB2014I88-1HS*	MG971356	*Nocardia cummidelens strain AQ11*	MF928385.1	99	98
			*Nocardia sp. LC057*	JQ014421.1		
			*Nocardia cummidelens strain DR02*	MF928296.1		
Okhotnichya	*Nocardia sp. IB2014I79-1*	MG971360	*Nocardia cummidelens strain AQ11*	MF928385.1	99	98
			*Nocardia cummidelens strain DR02*	MF928296.1		
			*Nocardia sp. JSZCL7*	KU643201.1		
	*Nocardia sp. IB2014I79-2HS*	MG971359	*Nocardia sp. DP_00094*	KM274110.1	98	99
			*Nocardia ignorata*	LN867132.1		
			*Nocardia soli strain en43*	KP137544.1		
	*Nocardia sp. IB2014I79-3HS*	MG971358	*Nocardia sp. 01-Gi-008*	GU574061.1	100	100
			*Nocardia soli strain KSI*	KC113164.1		
			*Nocardia cummidelens strain HBUM174688*	FJ532399.1		
	*Nocardia sp. IB2014I79-4*	MG971357	*Nocardia fluminea*	LN774198.1	98	99
			*Nocardia sp. QLS54*	JQ838098.1		
			*Nocardia sp. 01-Gi-008*	GU574061.1		
	*Nocardiopsis sp. IB2014I79-5*	MG971355	*Nocardiopsis dassonvillei subsp. albirubida NRC2AzA*	LC366927.1	99	99
			*Nocardiopsis dassonvillei subsp. albirubida strain OAct926*	MG661750.1		
			*Nocardiopsis dassonvillei subsp. albirubida strain VTT E-062983*	EU430536.1		

For phylogenetic analysis, the sequences were aligned using the MEGA software (version 7.0) (Kumar, Stecher & Tamura, 2016). The evolutionary history was inferred using the maximum parsimony method. The percentage of replicate trees, in which the associated taxa clustered together in the bootstrap test (1,000 replicates), is shown next to branches (Felsenstein, 1985).

### Cultivation and extraction

The isolated strains were cultivated in 30 mL of production medium in 250 mL shake flasks with baffles for 5 days at 28 °C at 180 rpm shaking rate. Four different liquid media were chosen to estimate the metabolite production and fatty acids content. All chemicals used in this research were manufactured by Sigma-Aldrich (St. Louis, MO, USA), MP-biomedicals (Illkirch, France), and Bacto (France). These media are: NL19 (soy flour – 20 g, D-mannitol – 20 g, tap water – 1 L, pH 7.2), ISP2 (yeast extract – 4 g, malt extract – 30 g, starch – 4 g, tap water – 1 L, pH 7.3), SGG (starch soluble – 10 g, glucose – 10 g, glycerol – 10 g, cornsteep powder– 2.5 g, bacto peptone – 5 g, yeast extract – 2 g, NaCl – 1 g, CaCO_3_ – 3 g, tap water – 1 L, pH 7.3). To compare production efficiency of secondary metabolites of strains cultivated in above rich media, we used minimal medium (MM) that contained only glucose as a carbon source. Composition of this medium is as follows: L-asparagine – 0.5 g, K_2_HPO4 – 0.5 g, MgSO_4_ × 7H_2_O – 0.2 g, FeSO_4_ × 7H_2_O – 0.01 g, glucose – 10 g, distilled water – 1 L, pH 7.0– 7.2).

#### Extraction of secondary metabolites

The grown cultures were centrifuged at 3,000 rpm for 10 min to separate the biomass and cultural liquid. Then, secondary metabolites were extracted from the cultural liquid with equal volume of ethyl acetate. To extract natural products from the biomass, we used 10 mL of acetone:methanol mixture (ratio 1:1). The extraction was performed during 1 h on a rotator at 100 rpm at room temperature. The obtained crude extracts were evaporated *in vacuo* using IKA RV-8 rotatory evaporator (IKA, Staufen, Germany) at 40 °C and dissolved in 0.5 uL of methanol (Axenov-Gribanov et al., 2016).

#### Extraction of fatty acids from cultural liquid

FA were extracted from cultural liquid according to the modified assay described in (Matyash et al., 2008). For this part of our study, we added 0.5 volume of extracting mixture MTBE:MeOH:H_2_O (ratio 10:3:2.5) to 1 volume of cultural liquid; the mixture was incubated in S4 Skyline shaker (Elmi, Latvia) for 1 h at room temperature and then centrifugated at 1,000 rpm for 10 min. The upper phase was transferred to the rotor evaporator flask. Then, we added 10% of extracting mixture to the lower phase, and extracted FA again. The obtained upper phase was combined with the first fraction and evaporated *in vacuo* at 40 °C using a rotatory evaporator. The concentrated extract in amount of 1–2 mL was transferred into glass vials. Rotor evaporator flasks were washed three times with the mixture of CHCl_3_:MeOH:H_2_O (ratio 60:30:5), and their content was transferred to glass vials with semidry extracts. Finally, the obtained mixture was totally evaporated using a flow nitrogen evaporator at 40 °C and dissolved in CHCl_3_:methanol:water (ratio 60:30:5) solution in final concentration 1 mg/100 uL.

#### Extraction of fatty acids from cell biomass

FA were extracted from cell biomass according to the modified assay described in (Lewis, Nichols & McMeekin, 2000). Bacterial cells obtained after centrifugation were frozen overnight at −80 °C. Then, the cells were defrosted and washed three times with 5–7 mL of 0.9% NaCl solution with simultaneous intensive shaking on vortex. Then, the samples were placed in a sonic bath for 10 min with further centrifugation at 3,000 g for 10 min. Supernatant was discarded each time to obtain the washed cell biomass. Then, the procedure of FA isolation was similar to the one described above for FA extraction from cultural liquid.

### Estimation of fatty acids composition

We used gas chromatography to analyze fatty acid composition of the total fatty acid extracts of isolated strains. Fatty acid methyl esters (FAMEs) were identified using a “Chromatek-Crystall-5000.2” with a 2D sample injector (Chromatek, Yoshkar-Ola, Russia) gas chromatograph with a flame-ionization detector and a Zebron ZB-FFAP capillary gas chromatographic column. An isothermal column configuration was used. The temperature of detector and evaporator was 240 °C. The internal standard was C 22:0 FA. Chromatek-Analytik-5000.2 software was used for data recording and integration. FAMEs were identified with standard mixtures Supelco 37 FAME mix (Sigma Aldrich, St. Louis, MO, USA) and by comparing the equivalent lengths of carbon chains and table constants according to (Cabrini et al., 1992; Gago et al., 2011).

### Antimicrobial activity assay of extracts from isolated strains

Antimicrobial activities of the extracted metabolites were tested using the disk diffusion method (Burdass, Grainger & Hurst, 2001). 100uL of bacterial and fungal 12-h test cultures were plated and dried on solid LB (for bacteria) and YPD (for fungi) media. Thirty uL of each crude extract dissolved in methanol was loaded on 5 mm diameter paper discs, and the disks were dried naturally. Paper disks loaded with 30uL of pure methanol were used as a negative control. Then, the disks were placed on solid LB or YPD agar media. The plates were incubated 12–24 h at 37 °C for bacteria and 30 °C for fungi (Pinheiro et al., 2018). Several bacterial and fungal test cultures, such as *Bacillus subtilis* ATCC 66337, *Staphylococcus carnosus* ATCC 51365, *Pseudomonas putida* KT 2440, *Escherichia coli* ATCC25922, *Saccharomyces cerevisiae* BY4742 and *Candida albicans* DSM1665 were chosen to test antibiological properties. The test cultures were obtained from Leibniz-Institute DSMZ-German Collection of Microorganisms and Cell Cultures (*Braunschweig ,* Germany). The activity against *C. albicans* was estimated in intergovernmental veterinary laboratory of the federal service for veterinary and phytosanitary supervision (Irkutsk). The zones of inhibition around paper disks were measured manually with accuracy ±1 mm.

## Results

### Isolation and phylogenetic analysis of actinobacteria from water surface of underground lakes in Siberia

A total of 17 culturable actinobacteria strains were isolated from water surface of underground lakes in Badzheyskaya and Okhotnichya caves based on their morphological characteristics. Twelve out of 17 strains were isolated from Badzheyskaya cave, and the other five strains were isolated from Okhotnichya cave (Table 2).

**Figure 1:**
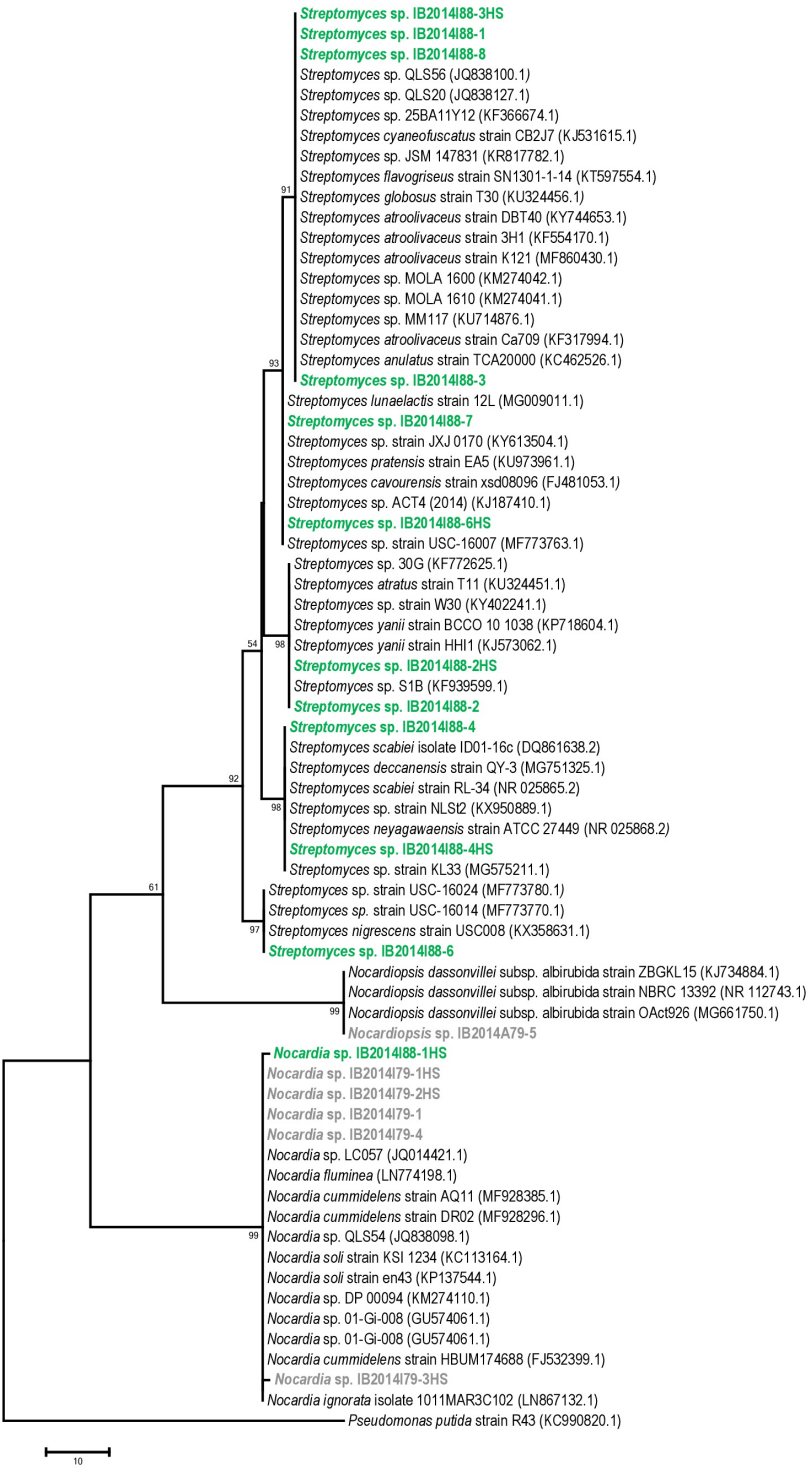
Maximum parsimony analysis of strains isolated from Badzheyskaya and Okhotnichya caves. The evolutionary history was inferred using the Maximum Parsimony method. Tree #1 out of 10 most parsimonious trees (length = 169) is shown. The consistency index is (0,829060), the retention index is (0,983065), and the composite index is 0,866726 (0,815020) for all sites and parsimony-informative sites (in parentheses). The percentage of replicate trees in which the associated taxa clustered together in the bootstrap test (1,000 replicates) are shown next to the branches. The MP tree was obtained using the Subtree-Pruning-Regrafting (SPR) algorithm with search level 1 in which the initial trees were obtained by the random addition of sequences (10 replicates). The tree is drawn to scale, with branch lengths calculated using the average pathway method and are in the units of the number of changes over the whole sequence. The analysis involved 70 nucleotide sequences. All positions containing gaps and missing data were eliminated. There were a total of 404 positions in the final dataset. Evolutionary analyses were conducted in MEGA7. Supplement: **Green & bold strains**–strains isolated from Badzheyskaya cave; **Gray & bold strains**–strains isolated from Okhotnichya cave.

The 16S rRNA gene sequence-based phylogenetic analysis revealed that 10 out of 11 strains isolated from Badzheyskaya cave belonged to the genus *Streptomyces*. Also, we isolated one strain of the *Nocardia* genus from this cave. Five other strains were isolated from Okhotnichya cave and they belonged to *Nocardia* and *Nocardiopsis* genus. Thus, in Badzheyskaya cave, a group of actinobacteria that belonged to Streptomycetaceae family was found as a dominant group of culturable actinobacteria, unlike in Okhotnichya cave, where the dominant group of culturable actinobacteria was presented by representatives of Nocardiaceae and Nocardiopsaceae families. As Fig. 1 shows, the obtained actinobacterial isolates are clustered with reference sequences of related species. Some of our strains (*Streptomyces* sp. IB2014I88-6HS and *Streptomyces* sp. IB2014I88-7) showed a close similarity to actinobacteria previously found in caves, such as *Streptomyces lunaelactis* strain (Table 1, Table S1, Fig. 1). This species was isolated from a moonmilk speleothem collected in Grotte des Collemboles’ (Comblain-au-Pont, Belgium) and described as a novel producer of ferroverdin A (Maciejewska et al., 2015). Another strain—*Streptomyces* sp. IB2014I88-4HS—showed a close similarity with *Streptomyces scabiei*. The latter is known as a phytopathogen (Bignell, Fyans & Cheng, 2014). All close representatives of *Nocardia sp.* were presented by nonpathogenic forms. A species close to the isolated strains of *Nocardiopsis* was *Nocardiopsis dassonvillei*. The latter is reported to be a rare infection agent for humans. This agent has been implicated in cutaneous, pulmonary, eye, nasal and disseminated infections (Bennur et al., 2015; Shivaprakash et al., 2012).

Also, we compared the sequences of the isolated strains with other representatives of *Streptomyces*, *Nocardia* and *Nocardiopsis* genera from other caves and water substrates. As Figs. S1–S3 show, our strains related to *Nocardia* and *Nocardiopsis* genera did not form tight different clades and were characterized by low similarity to other species registered in Ez Taxon database (Table S1). Representatives of *Streptomyces* strains isolated from Siberian caves form clades with other representatives of strains previously found in water sources (in case of *Streptomyces* sp. IB2014I88-6). Also, some of isolated strains form subclades in the tree. However, in general the absence of tight clades of isolated strains could be explained by ecology of isolated actinobacteria and their common distribution in environment (Schaechter, 2009).

### Analysis of biological activity of isolated strains

Antibiotic activities of the isolated strains are presented in Table 3 and Tables S2–S5*.* Ten (59%) out of 17 tested isolates showed antibiotic activity against at least one tested bacterial or fungal culture. The other seven (41%) strains (*Streptomyces* sp. IB2014I88-3HS, *Nocardia* sp.IB2014I88-1HS, *Nocardia* sp. IB2014I79-1, *Nocardia* sp. IB2014I79-2HS, *Nocardia* sp. IB2014I79-3HS, *Nocardia* sp. IB2014I79-4, and *Nocardiopsis* sp. IB2014I79-5) did not inhibit growth of any tested microorganisms under the employed conditions of cultivation. Among the seventeen isolates, only three strains (*Streptomyces* sp.IB2014I88-4, *Streptomyces* sp.IB2014I88-2HS and *Streptomyces* sp. IB 2014I88-1) grown in SGG and ISP2 media appeared to have a broad spectrum of antibiotic activity against all test organisms.

**Table 3:** Antibiotic activity of isolated strains grown on NL19, ISP2, SGG and MM media.

Strain	Medium	Test cultures					
		*B. subtilis*	*S. carnosus*	*E. coli*	*P. putida*	*S. cerevisiae*	*C. albicans*
*Streptomyces sp.* IB 2014I88-1	NL19						
	ISP2	CL, BM	CL, BM	CL, BM	CL, BM		CL
	SGG	CL	CL				CL
	MM	CL					CL
*Streptomyces sp.* IB 2014I88-2HS	NL19					BM	
	ISP2	CL, BM	CL, BM	CL, BM	CL, BM	CL, BM^a^	CL, BM
	SGG	CL	CL, BM	CL	CL	CL^a^, BM	BM
	MM						
*Streptomyces sp.* IB 2014I88-2	NL19						
	ISP2						
	SGG	CL	CL	CL	CL		CL
	MM						
*Streptomyces sp.* IB 2014I88-3	NL19					CL^a^, BM^a^	BM
	ISP2					BM^a^	
	SGG					CL^a^, BM	
	MM					BM	BM
*Streptomyces sp.* IB 2014I88-4HS	NL19	CL	CL	CL	CL		
	ISP2		CL				
	SGG						
	MM	CL					
*Streptomyces sp.* IB 2014I88-4	NL19	CL	CL, BM	CL, BM	CL, BM	CL	CL
	ISP2	CL, BM	CL, BM	CL, BM	CL, BM	CL^a^, BM	
	SGG	CL	CL, BM	CL, BM	CL, BM	CL^a^, BM^a^	CL, BM
	MM		CL, BM	CL, BM	CL, BM	CL	CL, BM
*Streptomyces sp.* IB 2014I88-6HS	NL19	CL				BM	
	ISP2						
	SGG						
	MM		BM	BM	BM		
*Streptomyces sp.* IB 2014I88-6	NL19						
	ISP2						
	SGG						
	MM	CL	CL	CL	CL		
*Streptomyces sp.* IB 2014I88-7	NL19		CL, BM	CL	CL		
	ISP2		BM		BM		
	SGG	CL	CL	CL	CL		
	MM						CL, BM
*Streptomyces sp.* IB 2014I88-8	NL19						
	ISP2						
	SGG	CL, BM	CL, BM	CL, BM	CL, BM		
	MM						

**Notes.**

CL cultural liquid extract BM biomass extract

a zone of inhibition more than 20 mm.

Eight (80%) out of ten mentioned active strains cultivated in the tested media inhibited growth of both Gram-positive and Gram-negative bacteria. Crude extracts obtained from the strains *Streptomyces* sp. IB2014I88-1, *Streptomyces* sp. IB2014I88-2HS, *Streptomyces* sp. IB2014I88-4 and *Streptomyces* sp. IB2014I88-7 were found to inhibit all Gram-positive and Gram-negative bacteria when they were grown in ISP2 medium. Five strains (*Streptomyces* sp. IB2014I88-2HS, *Streptomyces* sp. IB2014I88-2, *Streptomyces* sp. IB2014I88-4, *Streptomyces* sp. IB2014I88-7 and *Streptomyces* sp. IB2014I88-8) demonstrated inhibitory effects against *B. subtilis*, *S. carnosus*, *E. coli* and *P. putida* after cultivation in SGG medium. In addition, two strains—*Streptomyces* sp. IB2014I88-4HS and *Streptomyces* sp. IB2014I88-6—were active against all bacteria while cultivated in NL19 medium and MM medium, respectively.

One extract of the strain *Streptomyces* sp. IB2014I88-1 obtained from SGG medium was able to inhibit growth of all tested Gram-positive microorganisms but did not inhibit growth of Gram-negative bacteria. Growth of *B. subtilis* was hindered by three strains, including *Streptomyces* sp. IB2014I88-1 and *Streptomyces* sp. IB2014I88-4HS grown in MM medium and *Streptomyces* sp. IB2014I88-6HS grown in NL19 medium. We did not find specific ability of strains to inhibit growth of Gram-negative bacteria.

Eight out of ten mentioned active strains cultivated in the tested media inhibited growth of fungi. Four of them, including *Streptomyces* sp. IB2014I88-2HS, *Streptomyces* sp. IB2014I88-3, *Streptomyces* sp. IB2014I88-4HS and *Streptomyces* sp. IB2014I88-4 were active to inhibit growth of both *S. cerevisiae* and *C. albicans*. *Streptomyces* sp. IB2014I88-3 strain was able to inhibit growth of fungi but did not inhibit growth of bacteria under all tested conditions.

Along with cultivation of strains in nutrient-rich liquid media, growth of strains in poor MM media led to synthesis of fungicidal compounds. MM medium extracts obtained from three *Streptomyces* strains (*Streptomyces* sp. IB2014I88-3, *Streptomyces* sp. IB2014I88-7 and *Streptomyces* sp. IB2014I88-4) showed activity against yeasts. Also, some specific activity intended to hinder growth of *C. albicans* was found. Thus, strains *Streptomyces* sp. IB2014I88-1, *Streptomyces* sp. IB2014I88-2 and *Streptomyces* sp. IB2014I88-7 inhibited growth of pathogenic *C. albicans* but did not inhibit growth of *S. cerevisiae*. Also, a multiple activity of strains against fungi was observed in minimal nutrient media.

### Estimation of fatty acid composition in isolated strains

**Figure 2:**
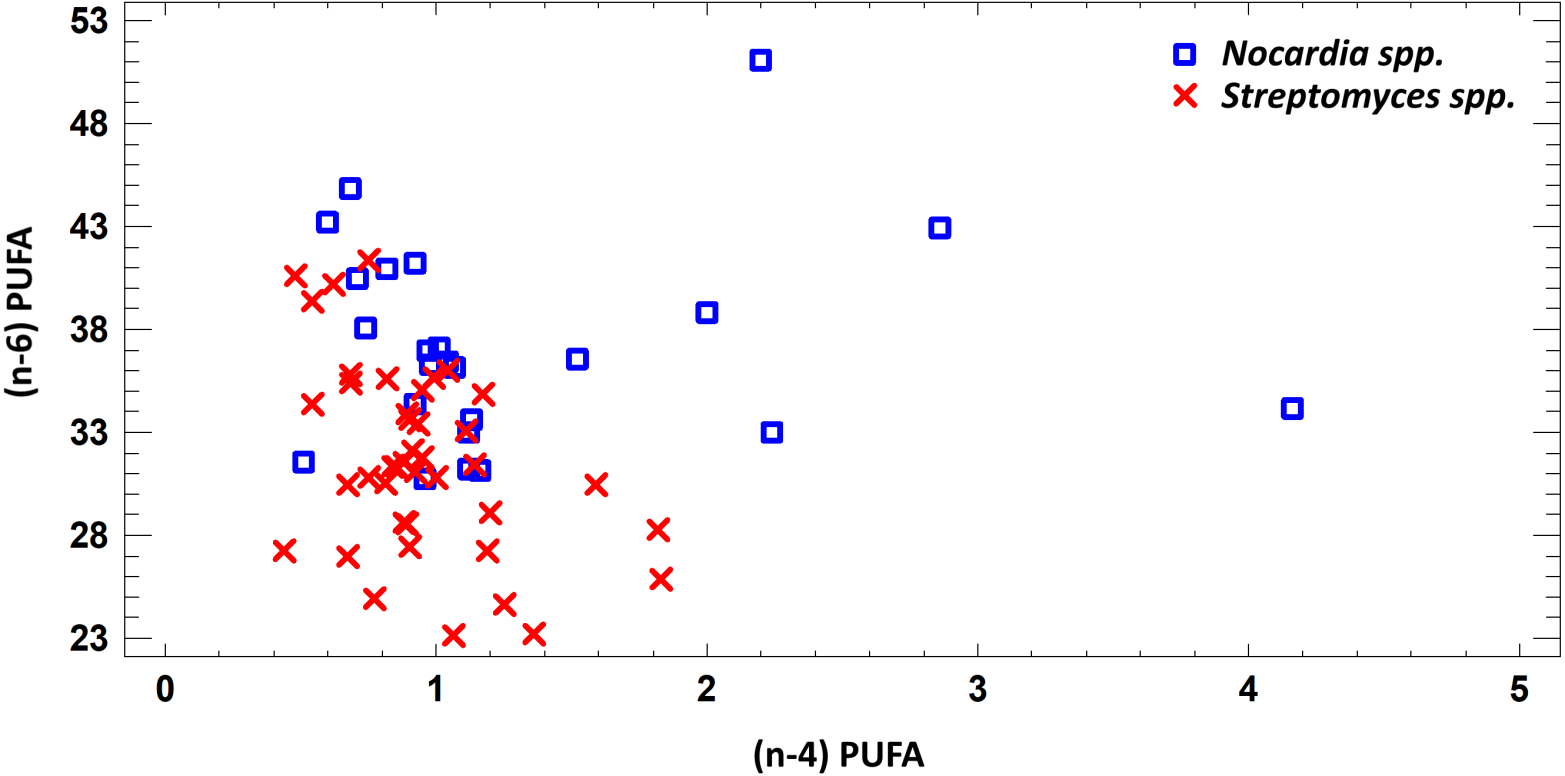
Differences of quantity of total (*n* − 4) and (*n* − 6) PUFA in cell biomass of *Nocardia* sp. and *Streptomyces* sp. isolated from underground lakes.

**Figure 3:**
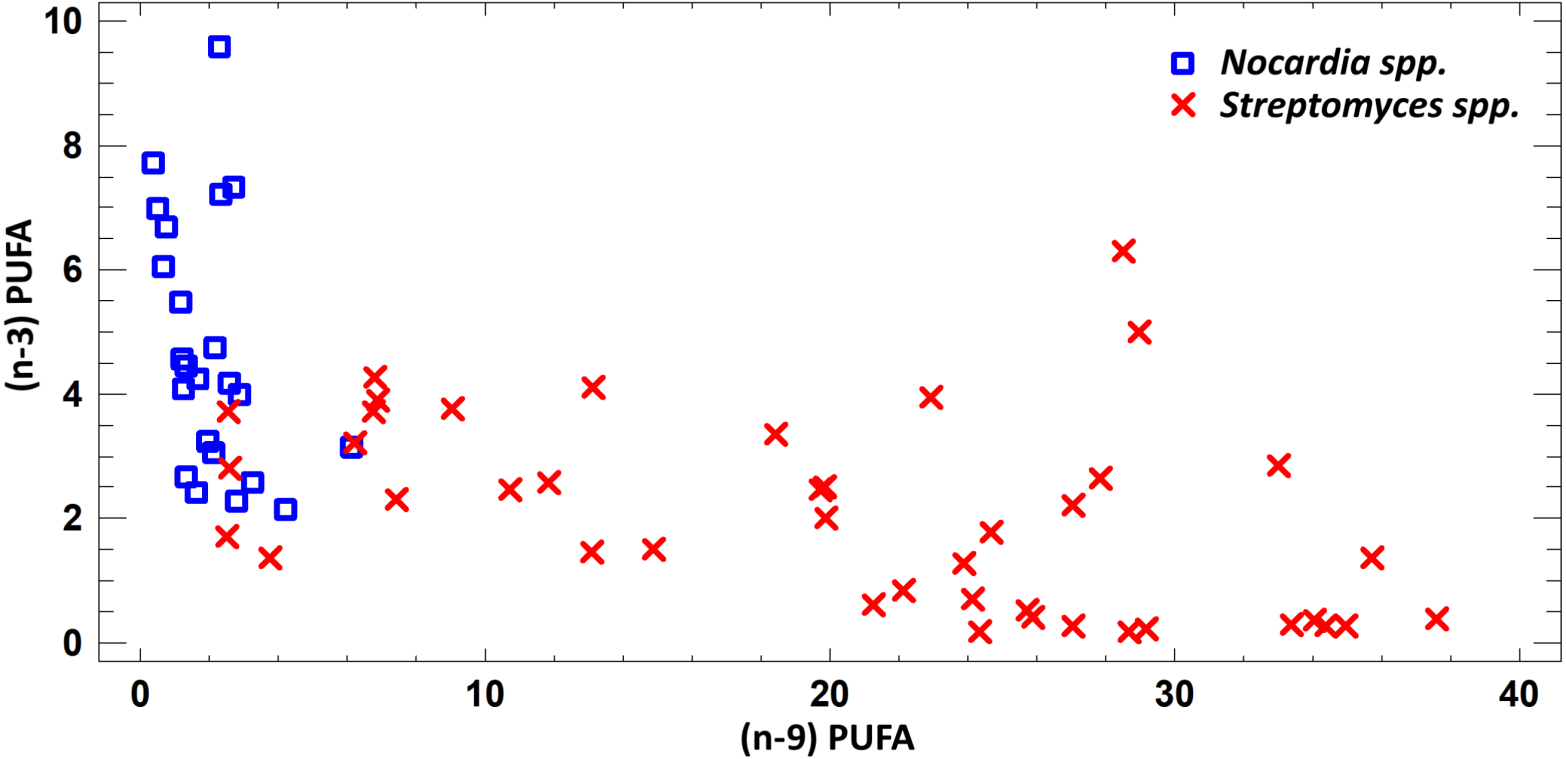
Assessment of (*n* − 9) and (*n* − 3) content of PUFA in cultural liquids of *Nocardia* and *Streptomyces*.

**Figure 4:**
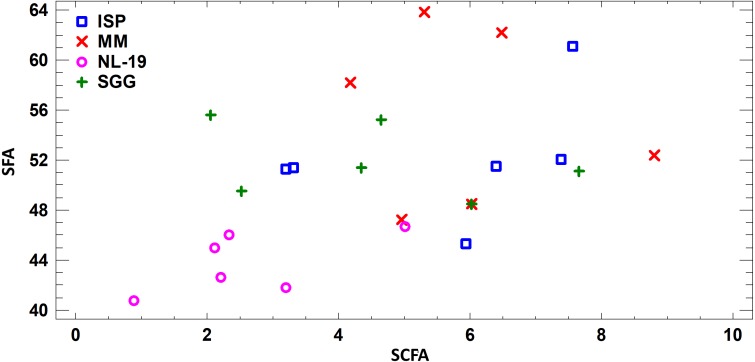
Differences of FA profiles of SCFA and SFA in cultural liquids of *Nocardia* from different nutrient media.

**Figure 5:**
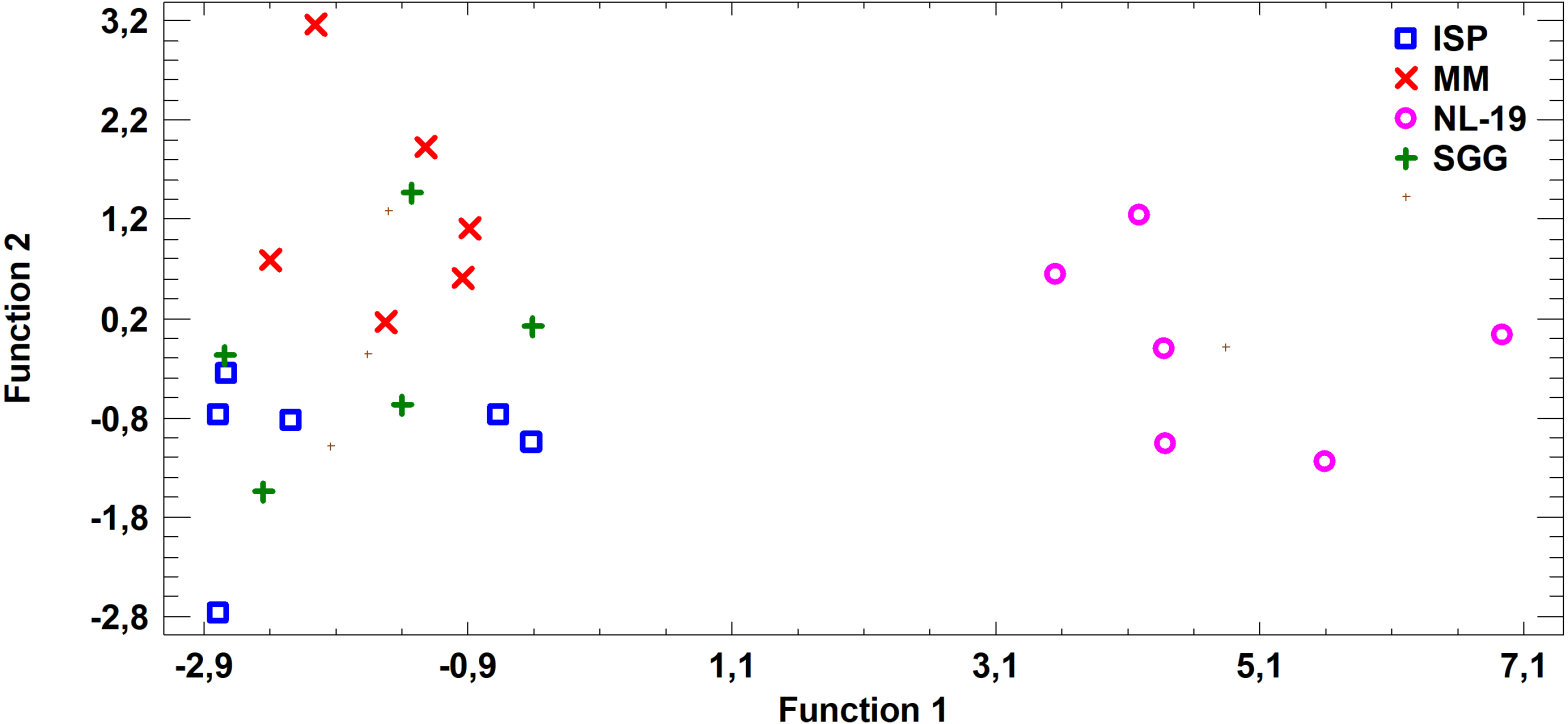
Discriminant analysis of FA profile based on major sums of different classes of FA (SCFA, SFA, MUFA, (*n* − 9) PUFA, (*n* − 7) PUFA, (*n* − 6) PUFA, (*n* − 4) PUFA, (*n* − 3) PUFA, PUFA) in cultural liquids of *Nocardia* after cultivating in different nutrient media.

**Figure 6:**
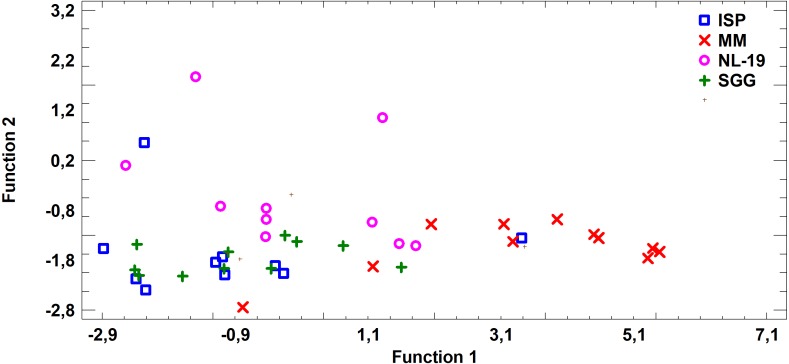
Discriminant analysis of FA profile based on major sums of different classes of FA (SCFA, SFA, MUFA, (*n* − 9) PUFA, (*n* − 7) PUFA, (*n* − 6) PUFA, (*n* − 4) PUFA, (*n* − 3) PUFA, PUFA) in cultural liquids of *Streptomyces* after cultivating in different nutrient media.

**Figure 7:**
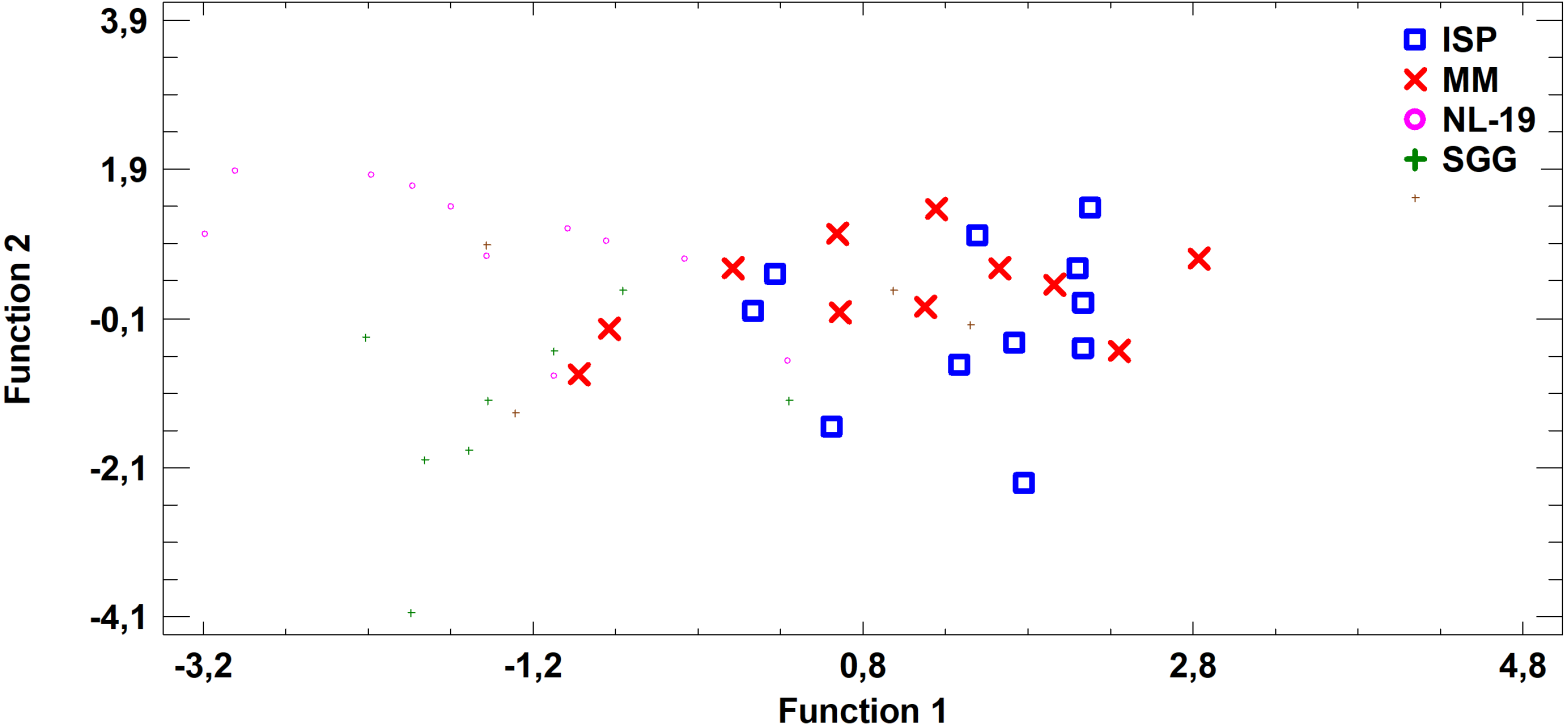
Discriminant analysis of FA profile based on major sums of different classes of FA (SCFA, SFA, MUFA, (*n* − 9) PUFA, (*n* − 7) PUFA, (*n* − 6) PUFA, (*n* − 4) PUFA, (*n* − 3) PUFA, PUFA) in biomass of *Streptomyces* after cultivating in different nutrient media.

In this study we estimated 60 parameters for each extract. Here, we present the summary data. When assessing the fatty acid (FA) profile, we found that polyunsaturated fatty acids (PUFA) were dominant quantitatively in the extracts of *Nocardia* and *Streptomyces* grown in different media. Saturated fatty acids (SFA) were the second most abundant type in the fatty acids profile. It was due to palmitic acid. Also, a few monounsaturated fatty acids (MUFA) were detected (Figs. 2–7; Table S6).

It was determined that fatty acid profiles of *Nocardia* and *Streptomyces* strains were significantly different. Primarily, it is linked to the cell wall composition of those microorganisms and their biosynthetic capabilities. Significant differences of total PUFA due to dominant class (*n* − 4) PUFA and minor (*n* − 6) PUFA were shown for extracts from cell biomass. Total amount of (*n* − 4) PUFA in cell biomass of *Nocardia* genera was 30.74–51.07%, while total amount of (*n* − 6) PUFA was 0.51–4.16%. Regarding representatives of *Streptomyces* genera, it was demonstrated that total amount of (*n* − 4) and (*n* − 6) was lower and varied in the range 23.12–41.33% for (*n* − 4) and in the range 0.44–1.83% for (*n* − 6). It was established that PUFA prevailed in the FA profile of the investigated bacteria. Besides, the fatty acids (*n* − 4) family prevailed in the PUFA because of C22:5 (*n* − 4). In terms of amount, SFAs took the second place (Fig. 2).

FA profile of cultural liquids of *Nocardia* and *Streptomyces* was characterized by SFA dominance. The SFA level for *Nocardia* was 40.75–63.85%, while for *Streptomyces* it was 17.22–62.69%. The second most abundant class was MUFA. MUFA levels in representatives of *Nocardia* sp. varied in the range 13.75–47.01% of overall fatty acid content. For *Streptomyces* spp. strains, it varied in the range 3.71–47.01% of the overall fatty acid content. Also, it should be noted that we detected significant amount of MUFA –C18:1 (*n* − 9), oleic acid, and C16:1(*n* − 7), palmitoleic acid. Figure 3 shows FA profile of cultural liquid of the isolated *Nocardia* and *Streptomyces* strains. Differences between those two genera were determined in terms of (*n* − 9) and (*n* − 3) PUFA (Fig. 3).

Assessing the fatty acid content in cultural liquid of *Nocardia* strain cultivated in different media, we found transgression of FA profiles. Thus, the FA profile obtained in case of bacterial growth in NL-19 medium significantly differs in terms of SFA and short chain fatty acids (SCFA) (Fig. 4).

FA profile of Nocardial cell walls grown on NL-19 medium was also different from FA profile of bacteria grown in other media (Fig. 5). Increased level of dominant (*n* − 4) PUFA as well as (*n* − 3) PUFA and (*n* − 6) PUFA was detected in that medium. At the same time, after analysis of FA levels of cultural liquid of *Streptomyces* it was established that the FA profile was greatly different as compared with samples grown in MM medium (Fig. 6).

Analysis of medium effects on the FA profile of cell biomass in *Streptomyces* revealed that bacteria growth on MM and ISP2 media is characterized by common FA profile in comparison to FA profile of bacteria cultured in NL-19 and SGG media. The latter pair has the minimal transgression (Fig. 7).

Thus, it was shown that despite the lack of antimicrobial and fungicidal activity of *Nocardia* extracts, those strains were exceptional in terms of their FA spectrum. *Nocardia* isolated from underground lakes of the caves contained high levels of PUFA from different classes, mainly (*n* − 4) and SFA, in cultural liquid. PUFA was dominant in cell biomass.

Even though many fatty acids possess antimicrobial activity, we cannot link antibiotic activity of the strains with the particular type of fatty acids. The activity of *Streptomyces* strains might be linked to both major and minor fatty acids including MUFA (Agoramoorthy et al., 2007; Gołębiowski et al., 2014). The most abundant monounsaturated fatty acids are palmitoleic and oleic acids. They are precursors of polyunsaturated fatty acids of the (*n* − 7) and (*n* − 9) families, respectively.

We found that oleic acid 18:1 (*n* − 9) prevailed in MUFA. The level of oleic acid ranged 11.89–19.88% of total FA in medium MM in the strains *Streptomyces* sp. IB2014I88-2, *Streptomyces* sp. IB2014I88-1, and *Streptomyces* sp. IB2014I88-3. It was the highest level of FA in comparison with other media. At the same time, concentration of oleic acid 18:1 (*n* − 9) was 18.58% of total FA in strain *Nocardia* sp. IB2014I79-3HS grown in SGG medium.

It should be noted that the cultural liquid of strains *Streptomyces* sp. IB2014I88-6HS and *Streptomyces* sp. IB2014I88-4HS grown on SGG medium contained another MUFA –palmitoleic acid, C16:1 (*n* − 7) in levels 22.04% and 19.91% of total FA, respectively. The high content (21.45%) of that fatty acid was noted for the *Streptomyces* sp. IB2014I88-4HS strain grown in MM medium.

During the present study it was shown that the obtained strains extracellularly synthesize linolenic acid C18:3 (*n* − 3) that is essential for humans. For the strains *Streptomyces* sp. IB2014I88-1HS, *Streptomyces* sp. IB2014I88-2HS, *Nocardia* sp. IB2014I79-1HS, *Nocardia* sp. IB2014I79-2HS and *Nocardia* sp. IB2014I79-3HS grown in NL-19 medium, the linolenic acid level was 23.70–26.19% of total FA; it was the highest level in comparison with other media. Besides, the level of linoleic acid in the strains *Streptomyces* sp. IB2014I88-2, *Streptomyces* sp. IB2014I88-3, *Nocardia* sp. IB2014I79-1, *Nocardia* sp. IB2014I79-4 grown in the same medium was 13.51–25.31% of total FA in comparison with other PUFA.

## Discussion

In this study we estimated the diversity of culturable actinobacteria strains that inhabit water surface of underground lakes in Badzheyskaya and Okhotnichya caves. This is the first study for those Siberian caves. Here, we analyzed their antimicrobial properties and fatty acids composition.

Dominance of culturable strains related to the genus *Streptomyces* in Badzheyskaya cave found in this study is not surprising, since previous investigations show high occurrence of this genus all over the world (Hamedi, Poorinmohammad & Wink, 2017) and especially in underground environments (De Leo et al., 2012; Nimaichand et al., 2015; Maciejewska et al., 2016). Members of this genus the *Streptomyces* genus are capable of using various mechanisms of competition including rapid limited-nutrient utilization combined with interference competition (Vaz Jauri et al., 2013; Schlatter & Kinkel, 2014). Also, they produce most of antibiotics and natural products used in pharmacy and medicine (Goodfellow & Fiedler, 2010; Bérdy, 2012; Riquelme et al., 2017).

However, the absence of culturable *Streptomyces* in Okhotnichya cave could be explained by specific hydrochemistry composition or high levels of iron or sulfur in the lake water, and low level of oxygen. The above data characterizing this hypothesis are not officially presented in the literature due to location and limited knowledge about this lake and its ecosystem. The fact that we isolated *Nocardiopsis* representatives in this water may indirectly confirm it. It is well known that this bacteria genus prevails as free-living entities in different ecosystems, including extreme conditions, such as hypersaline habitats on account of their salt-, alkali- and desiccation-resistant behavior (Bennur et al., 2015). Also, there is another open question driven by the paradox that in the water surfaces of underground lake in Okhotnichya cave (the cave with high recreation load) we did not find widely spread representatives of *Streptomyces*. As a preliminary hypothesis, this could be explained by unique microbial regulatory mechanisms of the caves, or microclimate parameters and the abiotic factors mentioned above.

Among the culturable diversity of Okhotnichya cave, genus *Nocardia* was found as a dominant. Also, a representative of *Nocardiopsis* genera was isolated. Isolation of *Nocardia and Nocardiopsis* strains has been previously mentioned for the cave environments (Groth et al., 1999; Jurado et al., 2010; Cheeptham et al., 2013; Jurado et al., 2014). It has been shown that *Nocardia* species inhabit both aquatic and terrestrial ecosystems (Bérdy, 2012). Representatives of the *Nocardia* and *Nocardiopsis* strains isolated in this study did not demonstrate any antibiotic activities *in vitro*. We suggest that it can be explained by inappropriate cultivation conditions of these genera, namely: short period of cultivation or media composition that resulted in low metabolic activity (Vartoukian, Palmer & Wade, 2010).

During the last 30 years, only few compounds have been isolated and identified from cave-dwelling actinobacteria. These compounds are: cervimycins A-D, xiakemycin A, hypogeamicin A, etc. Cervimycins are tetracyclic polyketides from *Streptomyces tendae*. They are characterized by antibacterial activities against multi-drug-resistant staphylococci and vancomycin-resistant enterococci (Herold et al., 2005). Xiakemycin A is a pyranonaphthoquinone antibiotic derived from *Streptomyces* sp. CC8-201 that possessed strong inhibitory activities against Gram-positive bacteria and showed cytotoxicity activity to a number of human cancer cells including lung cancer A549 cells, breast cancer MCF-7 cells, and hepatoma HepG-2 cells (Jiang et al., 2015). Hypogeamicin A presents a new S-bridged dimeric pyronaphthoquinone that was isolated from a rare actinobacteria strain *Nonomuraea specus* and demonstrated cytotoxic activity to the colon cancer derived cell line TCT-1 at low micromolar ranges (Derewacz et al., 2014b). Also, the strain *Streptomyces* sp. JS520 isolated from a cave in Serbia was able to produce undecylprodigiosin, which is characterized by antioxidative and UV-protective properties and inhibited growth of Gram-positive bacteria species and pathogenic *C. albicans* (Stankovic et al., 2012). M. Maciejewska with co-authors isolated actinobacteria of the genus *Streptomyces* from moonmilk deposits*.* These isolates inhibited growth of Gram-positive, Gram-negative bacteria and fungi (Maciejewska et al., 2016), and this activity was associated with activity of ferroverdin A.

Taking into consideration a limited number of elucidated compounds from cave-dwelling microbiota, there is a great number of metabolites whose chemical structure has not yet been determined (Tomova et al., 2013; Cheeptham et al., 2013). Also, in addition to antibiological activity it is important to estimate the ability of underground actinobacteria strains to produce FA. FA are aliphatic monobasic carboxylic acids that can be found in fat, oils, and waxes in etherified form. FA can be divided into three groups: saturated, monounsaturated, and polyunsaturated. SFA do not have double bonds, and they can be synthesized in animal body. They are myristinic acid (C14), palmitic acid (C16), stearic acid (C18). MUFA have one double bond, and they are essential because the desaturase enzyme participates in the synthesis of double bond. They are palmitoleic acid (C16:1) and oleic acid (C18:1). PUFA have more than one double bonds, and they are essential. They are arachidonic acid (C20:4(*n* − 6)), eicosapentaenoic acid (C20:5(*n* − 3)), docosahexaenoic acid (C22:6(*n* − 3)). PUFA are important for organisms for the following reasons: on the one hand, they modify physical characteristics of biological membranes adapting them to environmental conditions, and on the other hand, their oxidized derivatives regulate many cellular and tissue physiological processes.

The results of the present study demonstrate new producers for synthesis and extraction of *n* − 4, *n* − 7, *n* − 9 PUFA, which often have antimicrobial activity (Huang et al., 2010; Choi et al., 2013). Also, it should be noted that as previously mentioned by Georgel et al. (2005), antimicrobial activity of free fatty acids is significantly higher than the activity of natural antimicrobial peptides in vitro (Georgel et al., 2005). Thus, the obtained materials may become a basis for development of innovative approaches to utilize bacteria isolated from caves as a biological source of biologically active compounds to create medical and veterinary drugs.

## Conclusion

In this study we isolated 17 culturable actinobacteria strains collected in underground lakes of Badzheyskaya and Okhotnichya caves. We analyzed their actinobacterial diversity and found the absence of Streptomycetes strains in the underground lake of Okhotnichya cave that could be determined by specificities of ecosystem. Also, we estimated antimicrobial properties and composition of bacterial fatty acids under different cultivation conditions. We showed multiple antibacterial and antifungal activities of the isolated *Streptomyces* strains and the absence of antimicrobial activity of rare strains in tested conditions. At the same time, despite the lack of antimicrobial and fungicidal activity of *Nocardia* extracts, those strains characterized by ability of extracellularly synthesized linolenic acid that is essential for humans. *Nocardia* sp. isolated from cave underground lakes contained high levels of PUFA from different classes, mainly (*n* − 4) and SFA in cultural liquid as well as dominant PUFA in biomass. The quantitative content of FA in cultural liquid of isolated strains demonstrated that the class of polyunsaturated fatty acids prevailed over the saturated fatty acids, and monounsaturated fatty acids.

Thus, this is the first study of cultivated actinobacteria for those caves, where we showed that strains of actinobacteria isolated from water surface of underground lakes represent a promising source for development of novel drugs, and these results are highly pertinent in the light of global problems caused by development and spread of antibiotic resistance.

##  Supplemental Information

10.7717/peerj.5832/supp-1 Supplemental Information 1 Raw data of profile of fatty acids content

10.7717/peerj.5832/supp-2 Supplemental Information 2 Raw data of sum of fatty acids content

10.7717/peerj.5832/supp-3 Supplemental Information 3 Supplementary materials

10.7717/peerj.5832/supp-4 Supplemental Information 4 Raw data of resulting tables for fatty acids composition mentioned in the manuscript

10.7717/peerj.5832/supp-5 Supplemental Information 5 Raw data of different fatty acids content

10.7717/peerj.5832/supp-6 Supplemental Information 6 Photo of water in the cave

10.7717/peerj.5832/supp-7 Supplemental Information 7 The corridor in the cave

10.7717/peerj.5832/supp-8 Supplemental Information 8 Photo of cave: lake

## Abbreviations

MTBE: methyl-tert-butyl ether
OSMAC: one strain-many compounds approach
FA: fatty acids
SFA: saturated fatty acids
MUFA: monounsaturated fatty acids
SCFA: short chain fatty acids
FAMEs: fatty acid methyl esters

## Additional Information and Declarations

### DNA Deposition

The following information was supplied regarding the deposition of DNA sequences: Data was deposited at NCBI GenBank with accession numbers:
MG971353, MG971351, MG971352, MG971349, MG971350, MG971348, MG971354, MG971346, MG971347, MG971345, MG971344, MG971356, MG971360, MG971359, MG971358, MG971357, MG971355.

### Data Availability

The following information was supplied regarding data availability: The raw data is available as Supplemental Files.
