# Supplementary material for: Estimation of antimicrobial activities and fatty acid composition of actinobacteria isolated from water surface of underground lakes from Badzheyskaya and Okhotnichya caves in Siberia
Source: PeerJ. 2018 Oct 25;6:e5832. doi: 10.7717/peerj.5832 (PMC6204239; doi:10.7717/peerj.5832)
Supplement: Supplemental Information 3 [file peerj-06-5832-s003.docx]

**SUPPLEMENTARY MATERIALS**

**Estimation of antimicrobial activities and fatty acid composition of actinobacteria isolated from water surface of underground lakes from Badzheyskaya and Okhotnichya caves in Siberia**

Irina V. Voytsekhovskaya^1§^, Denis V. Axenov-Gribanov^1,2*§^, Svetlana A. Murzina^3^, Svetlana N. Pekkoeva^3^, Eugeniy S. Protasov^1^, Stanislav V. Gamaiunov^1^, Maxim A. Timofeyev^1^

^1^ Irkutsk State University, Irkutsk, Russia,

^2^ Baikal Research Centre, Irkutsk, Russia

^3^ Institute of Biology of the Karelian Research Centre of the Russian Academy of Sciences, Petrozavodsk, Karelia, Russia

**Figure S1. Maximum Parsimony analysis of *Streptomyces* taxa**

The evolutionary history was inferred using the Maximum Parsimony method. Tree #1 out of 4 most parsimonious trees (length = 151) is shown. The consistency index is ( 0.600000), the retention index is ( 0.910781), and the composite index is 0.766021 ( 0.546468) for all sites and parsimony-informative sites (in parentheses). The percentage of replicate trees in which the associated taxa clustered together in the bootstrap test (1000 replicates) are shown next to the branches. The MP tree was obtained using the Subtree-Pruning-Regrafting (SPR) algorithm with search level 1 in which the initial trees were obtained by the random addition of sequences (10 replicates). The tree is drawn to scale, with branch lengths calculated using the average pathway method and are in the units of the number of changes over the whole sequence. The analysis involved 65 nucleotide sequences. All positions containing gaps and missing data were eliminated. There were a total of 477 positions in the final dataset. Evolutionary analyses were conducted in MEGA7. Supplement: Blue strains – strains isolated from water sources; Black strains – strains isolated from cave sources and outgroup; **Green & bold strains** – strains isolated from Badzheyskaya cave.

***Streptomyces* sp. IB2014I88-6HS (MG971346.1)**

*Streptomyces sp. strain AC390 FSS594 (KX928432.1)*

*Streptomyces lunaelactis* strain MM109 (KJ862793.1

*Streptomyces lunaelactis* strain MM25 (KJ862784.2)

*Streptomyces lunaelactis* strain MM29 (KJ862786.2)

*Streptomyces lunaelactis* strain MM32 (KJ862788.2)

*Streptomyces lunaelactis* strain MM40 (KJ862790.2)

*Streptomyces lunaelactis* strain MM93 (KJ862792.2)

*Streptomyces lunaelactis* strain MM115 (KJ862795.2)

*Streptomyces lunaelactis* strain MM109 (NR 134822.1)

*Streptomyces* sp. AKB-2008-ET11 (AM988883.1)

*Streptomyces flavofuscus (LT899925.1)*

*Streptomyces* sp. strain AC395 FSS602 (KX928437.1)

*Streptomyces lunaelactis* strain MM15 (KJ862783.1)

*Streptomyces lunaelactis* strain MM28 (KJ862785.2)

*Streptomyces lunaelactis* strain MM31 (KJ862787.2)

*Streptomyces lunaelactis* strain MM37 (KJ862789.2)

*Streptomyces lunaelactis* strain MM91 (KJ862791.2)

*Streptomyces lunaelactis* strain MM113 (KJ862794.2)

*Streptomyces lunaelactis* strain MM109 (KM207217.2)

***Streptomyces sp. IB2014I88-7 (MG971345.1)***

*Streptomyces* sp. strain ICC13 (KY419872.1)

*Streptomyces* sp. strain AC388 FSS592 (KX928430.1)

*Streptomyces cirratus* strain ICC15 (KY419852.1)

*Streptomyces* sp. GGC-D15 (FJ348016.1)

*Streptomyces* sp. cc8-201 (KJ480796.1)

*Streptomyces* sp. strain ICC4 (KY419849.1)

*Streptomyces* sp. PJF3 (KC978876.1)

*Streptomyces* sp. GGC-D17D (FJ348034.1)

***Streptomyces* sp. IB2014I88-1 (MG971353.1)**

***Streptomyces* sp. IB2014I88-8 (MG971344.1)**

***Streptomyces* sp. IB2014I88-3HS (MG971349.1)**

***Streptomyces* sp. IB2014I88-3 (MG971350.1)**

*Streptomyces cyaneogriseus* (FM998727.1)

*Streptomyces* sp. strain AC387 FSS590 (KX928429.1)

*Streptomyces* sp. strain AC386 FSS589 (KX928428.1)

*Streptomyces microflavus* (HF952703.1)

*Streptomyces* sp. strain M1-MRL (KX371098.1)

*Streptomyces* sp. A-189 (LT907818.1)

*Streptomyces hygroscopicus* strain SM2 (MF784462.1)

*Streptomyces* sp. A3a-6 (HG738950.1)

***Streptomyces* sp. IB2014I88-2HS (MG971351.1)**

***Streptomyces* sp. IB2014I88-2 (MG971352.1)**

*Streptomyces* sp. strain AC382 FSS585 (KX928424.1)

*Streptomyces* sp. strain AC378 FSS581 (KX928420.1)

***Streptomyces* sp. IB2014I88-4HS (MG971348.1)**

***Streptomyces* sp. IB201488-4 (MG971354.1)**

*Streptomyces scabiei* strain BCCO 10 524 (KP718596.1)

*Streptomyces nigrescens* strain USC008 (KX358631.1)

***Streptomyces* sp. IB2014I88-6 (MG971347.1)**

*Streptomyces* sp. PJG4 (KC978877.1)

*Streptomyces geysiriensis* strain USC016 (KX358639.1)

*Streptomyces* sp. strain AC381 FSS584 (KX928423.1)

*Streptomyces* sp. strain AC393 FSS600 (KX928435.1)

*Streptomyces* sp. strain AC394 FSS601 (KX928436.1)

*Streptomyces* sp. strain AC372 FSS574 (KX928415.1)

*Streptomyces* sp. A-191 (LT907819.1)

*Streptomyces* sp. strain AC380 FSS583 (KX928422.1)

*Streptomyces* sp. strain AC384 FSS587 (KX928426.1)

*Streptomyces collinus* strain ICN1 (JN187857.1)

*Streptomyces* sp. K11-0400 (LC322264.1)

*Streptomyces* sp. Alex. SK-121 (KJ726667.1)

*Streptomyces collinus* strain ICN2 (JN187858.1)

*Streptomyces albus* strain IMS1 (KY942141.1)

*Pseudomonas putida (KC990820.1)*

84

94

79

92

68

86

65

57

91

62

67

75

66

10

**Figure S2. Maximum Parsimony analysis of *Nocardia* taxa**

The evolutionary history was inferred using the Maximum Parsimony method. The most parsimonious tree with length = 197 is shown. The consistency index is ( 0,673267), the retention index is ( 0,921241), and the composite index is 0,766921 ( 0,620241) for all sites and parsimony-informative sites (in parentheses). The percentage of replicate trees in which the associated taxa clustered together in the bootstrap test (1000 replicates) are shown next to the branches. The MP tree was obtained using the Subtree-Pruning-Regrafting (SPR) algorithm with search level 1 in which the initial trees were obtained by the random addition of sequences (10 replicates). The tree is drawn to scale , with branch lengths calculated using the average pathway method and are in the units of the number of changes over the whole sequence. The analysis involved 36 nucleotide sequences. All positions containing gaps and missing data were eliminated. There were a total of 497 positions in the final dataset. Evolutionary analyses were conducted in MEGA7. Supplement: Blue strains – strains isolated from water sources; Black strains – strains isolated from cave sources and outgroup; **Green & bold strains** – strains isolated from Badzheyskaya cave; **Gray & bold strains** – strains isolated from Okhotnichya cave.

*Nocardia* sp. IB2014I100-5 (KT266902.1)

*Nocardia* sp. IB2014I79-1HS (KT266896.1)

***Nocardia* sp. IB2014I79-3HS (MG971358.1)**

*Nocardia* sp. IB2014I100-1HS (KT266903.1)

***Nocardia* sp. IB2014I79-4 (MG971357.1)**

*Nocardia* sp. ALBR8S4 (KP836264.1*)*

*Nocardia fluminea* strain ALCAN5S3 (KP836257.1)

*Nocardia* sp. JSZCL7 (KU643201.1)

***Nocardia* sp. IB2014I79-2HS (MG971359.1)**

***Nocardia* sp. IB2014I88-1HS (MG971356.1)**

*Nocardia* sp. IB2014I79-1HS (KT266896.1)

***Nocardia* sp. IB2014I79-1 (MG971360)**

*Nocardia* sp. 15-Je-015 (GU574172.1)

*Nocardia* sp. UA-VH0212 (KF577506.1)

*Nocardia* sp. LC001 (JQ014384.1)

*Nocardia jejuensis* strain N3-2 (AY964666.1)

*Nocardia* sp. LC520 (JQ014340.1)

*Nocardia altamirensis* strain DSM 44997 (EU006090.1)

*Nocardia* sp. NCCP-1342 (LC065369.1)

*Nocardia* sp. NN256 (GU723668.1)

*Nocardia asteroids* (FR799430.1)

*Nocardia* sp. NCCP-1173 (LC065200.1)

*Nocardia* sp. 11-Je-002 (GU574168.1)

*Nocardia* sp. 06-Be-019 (GU574165.1)

*Nocardia* sp. 12A-Be-004 (GU574169.1)

*Nocardia* sp. 12B-Be-002 (GU574170.1)

*Nocardia* sp. 05-St-015 (GU574164.1)

*Nocardia* sp. 08-St-007 (GU574167.1)

*Nocardia* sp. 03-St-022 (GU574163.1)

*Nocardia* sp. 07-Be-006 (GU574166.1)

*Nocardia* sp. 03-St-014 (GU574162.1)

*Nocardia* sp. 14-Be-019 (GU574171.1)

*Nocardia* sp. 03-St-006-Luft (GU574161.1)

*Nocardia* sp. W1.09-127 (JX458417.1)

*Nocardia* sp. strain ICC14 (KY419851.1)

*Pseudomonas putida* strain R43 (KC990820.1)

92

68

87

67

96

90

96

100

95

98

84

95

10

**Figure S3. Maximum Parsimony analysis of *Nocardiopsis* taxa**

The evolutionary history was inferred using the Maximum Parsimony method. Tree #2 out of 2 most parsimonious trees (length = 187) is shown. The consistency index is ( 0.656250), the retention index is ( 0.828125), and the composite index is 0.730699 ( 0.543457) for all sites and parsimony-informative sites (in parentheses). The percentage of replicate trees in which the associated taxa clustered together in the bootstrap test (1000 replicates) are shown next to the branches. The MP tree was obtained using the Subtree-Pruning-Regrafting (SPR) algorithm with search level 1 in which the initial trees were obtained by the random addition of sequences (10 replicates). The tree is drawn to scale, with branch lengths calculated using the average pathway method and are in the units of the number of changes over the whole sequence. The analysis involved 30 nucleotide sequences. All positions containing gaps and missing data were eliminated. There were a total of 502 positions in the final dataset. Evolutionary analyses were conducted in MEGA7. Supplement: Blue strains – strains isolated from water sources; Black strains – strains isolated from cave sources and outgroup; **Green & bold strains** – strains isolated from Badzheyskaya cave; **Gray & bold strains** – strains isolated from Okhotnichya cave.

*Nocardiopsis* sp. strain YIM C01603 (MF431341.1)

*Nocardiopsis* sp. strain YIM C01549 (MF431317.1)

*Nocardiopsis* sp. *G143 (JN633957.1)*

*Nocardiopsis* sp. Al-H10-3 (KF384498.1)

*Nocardiopsis* sp. strain YIM C01468 (MF431289.1)

*Nocardiopsis alba* strain S-2 (KF543078.1)

*Nocardiopsis* sp. strain M4 9 (MG758019.1)

*Nocardiopsis dassonvillei* strain OK-22 (KF543091.1)

*Nocardiopsis lucentensis* strain OK-2 (*KC759324.1*)

***Nocardiopsis* sp. IB2014A79-5 (MG971355.1)**

*Nocardiopsis dassonvillei* strain OK-1 (KF543079.1)

*Nocardiopsis* sp. OK-19 (KF543090.1)

*Nocardiopsis* sp. OK-23 (KF543089.1)

*Nocardiopsis* sp. OK-17 (KF543087.1)

*Nocardiopsis* sp. D-8 (KF543081.1)

*Nocardiopsis* sp. OK-20 (KF543086.1)

*Nocardiopsis* sp. OK-13 (KF543092.1)

*Nocardiopsis* sp. D-2 (KF543082.1)

*Nocardiopsis dassonvillei* (KF543085.1)

*Nocardiopsis sp.* strain YIM C01537 (MF431314.1)

*Nocardiopsis dassonvillei* strain OK-8 (KF543088.1)

*Nocardiopsis* sp. OK-24 (KF543084.1)

*Nocardiopsis sp. OK-6 (KF543083.1)*

*Nocardiopsis* sp. OK-10 (KF543080.1)

*Nocardiopsis alba* strain MML2606 (KT315905.1)

*Nocardiopsis* sp. MML2612 (KF734437.1)

*Nocardiopsis akesuensis* strain TRM 46250 (JX244077.1)

*Nocardiopsis akesuensis* strain TRM 46250 (NR 153668.1)

*Nocardiopsis akesuensis* strain TRM 46250 (KU860457.1)

*Pseudomonas putida* strain R43 (KC990820.1)

93

88

99

58

96

76

92

10

**Table S1** Actinobacteria strains isolated from water of underground lakes in Badzheyskaya and Okhotnichya caves and characterized by Ez Taxon database.

**Table S2** Antibiotic activity of the biomass and culture liquid extracts of culturable actinobacteria grown in NL-19 medium

*Disk diameter – 5 mm

->number – bacteriostatic activity

Number – diameter of inhibition zone (mm)

**Table S3** Antibiotic activity of biomass and culture liquid extracts of culturable actinobacteria grown in ISP2 medium

*Disk diameter – 5 mm

->number – bacteriostatic activity

Number – diameter of inhibition zone (mm)

**Table S4** Antibiotic activity of biomass and culture liquid extracts of culturable actinobacteria grown in SGG medium

*Disk diameter – 5 mm

->number – bacteriostatic activity

Number – diameter of inhibition zone (mm)

**Table S5** Antibiotic activity of biomass and culture liquid extracts of culturable actinobacteria grown in Minimal medium

*Disk diameter – 5 mm

->number – bacteriostatic activity

Number – diameter of inhibition zone (mm)

**Table S6** Summe and distribution of fatty acid composition of *Streptomyces* and *Nocardia* strains isolated from underground lakes

Legend: CL – cultural liquid extract; BM – biomass extract.

The values mean the percent of mentioned fatty acid from total amount of fatty acids composition
