# Supplementary figures and images for: Estimation of antimicrobial activities and fatty acid composition of actinobacteria isolated from water surface of underground lakes from Badzheyskaya and Okhotnichya caves in Siberia
Source: PeerJ. 2018 Oct 25;6:e5832. doi: 10.7717/peerj.5832 (PMC6204239; doi:10.7717/peerj.5832)

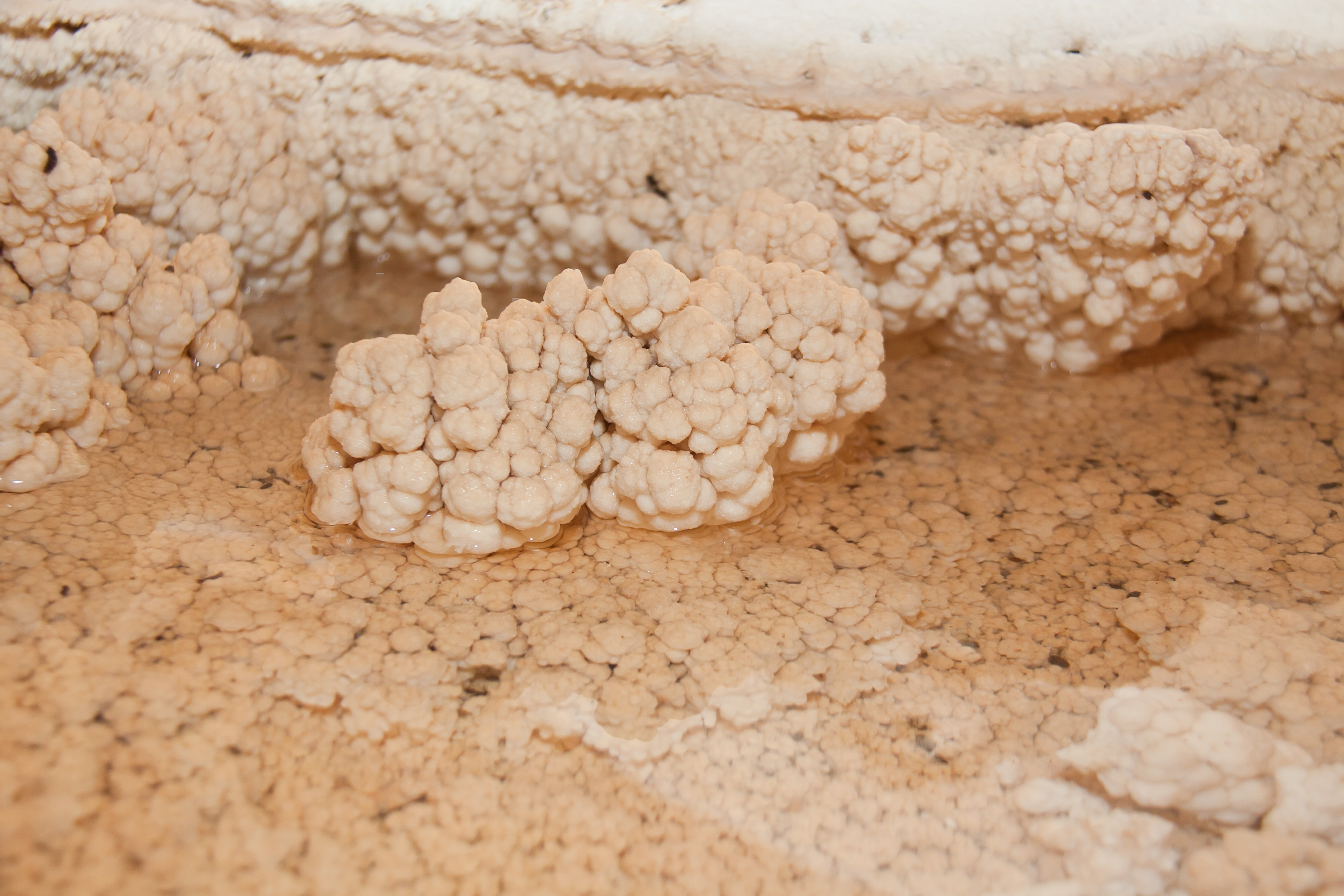

Supplement: Supplemental Information 6 [file peerj-06-5832-s006.jpg]

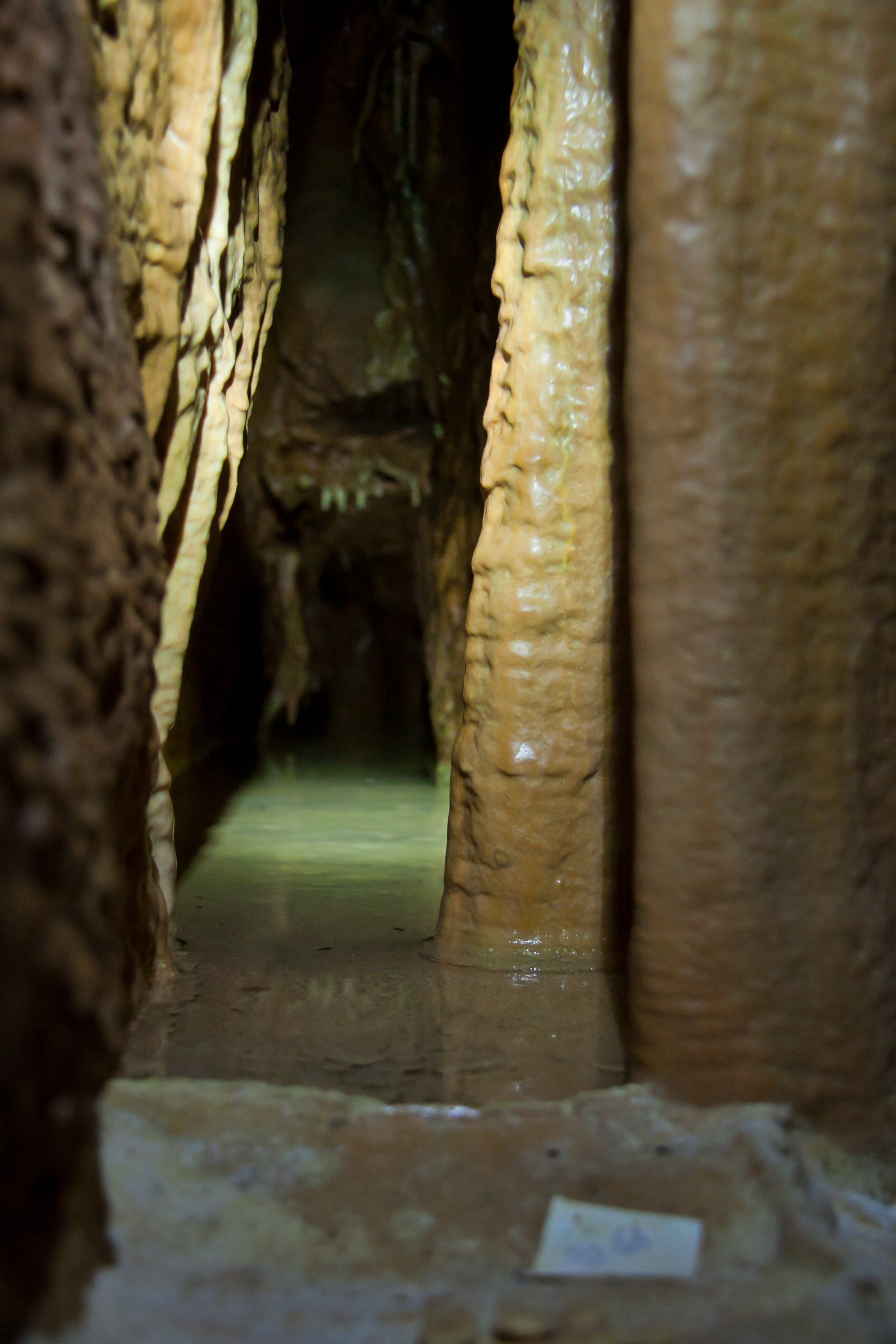

Supplement: Supplemental Information 7 [file peerj-06-5832-s007.jpg]

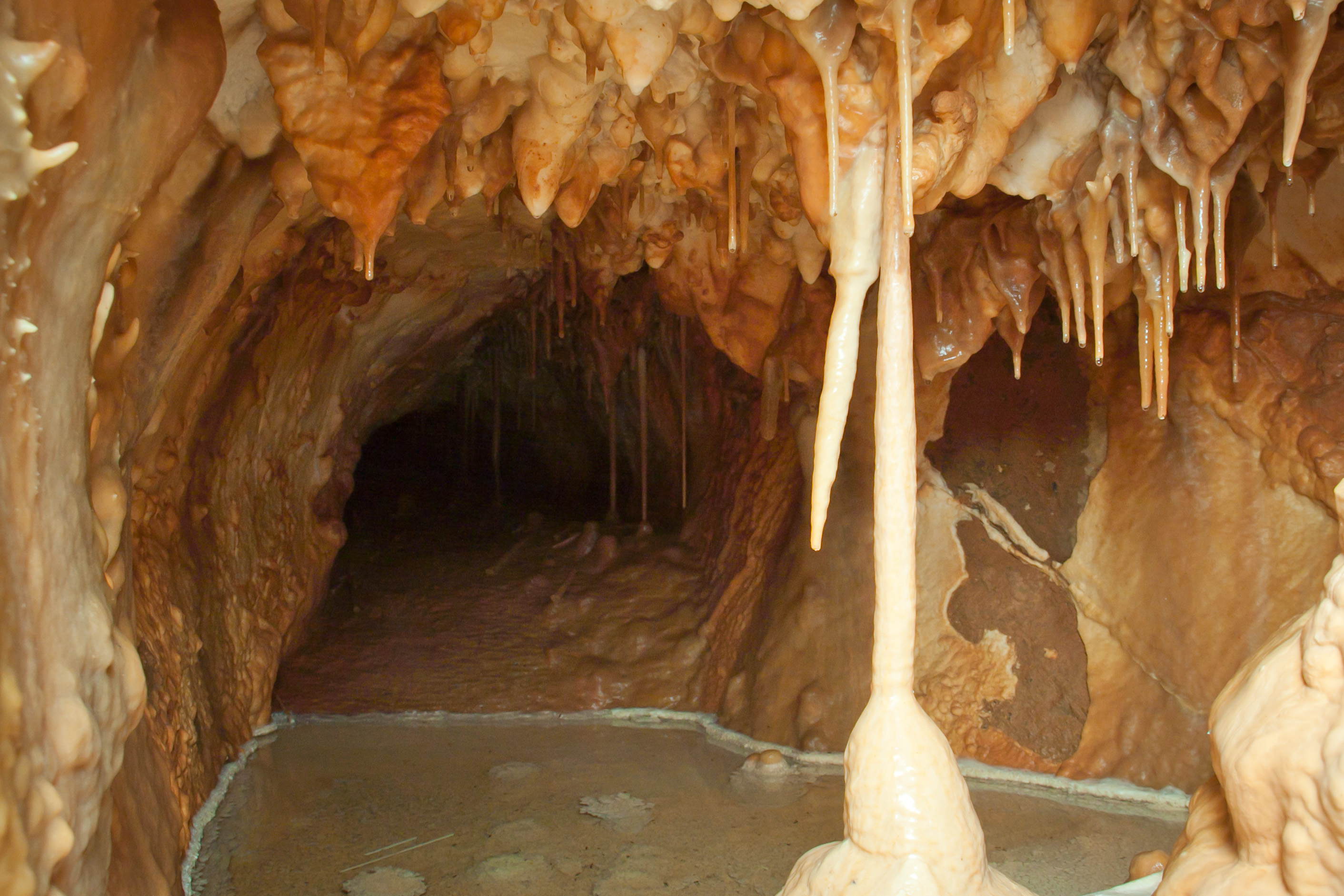

Supplement: Supplemental Information 8 [file peerj-06-5832-s008.jpg]
